# Supplementary material for: Perturbing proteomes at single residue resolution using base editing
Source: Nat Commun. 2020 Apr 20;11:1871. doi: 10.1038/s41467-020-15796-7 (PMC7170841; doi:10.1038/s41467-020-15796-7)
Supplement: Supplementary file 5 — Description of Additional Supplementary Files [file 41467_2020_15796_MOESM5_ESM.pdf]

Title: Supplementary Dataset 1
Description: gRNA sequences targets, predicted outcomes, scores, and other relevant annotations.

Title: Supplementary Dataset 2
Description: Supplementary Table 7 (Oligonucleotides used in this study)

Title: Supplementary Dataset 3
Description: Supplementary 8 (Media recipes used in this study).
