# Supplementary material for: Perturbing proteomes at single residue resolution using base editing
Source: Nat Commun. 2020 Apr 20;11:1871. doi: 10.1038/s41467-020-15796-7 (PMC7170841; doi:10.1038/s41467-020-15796-7)
Supplement: Supplementary file 6 — Reporting Summary [file 41467_2020_15796_MOESM6_ESM.pdf]

nature research

Corresponding author(s): Christian R Landry

Last updated by author(s): Mar 18, 2020

Reporting Summary

Nature Research wishes to improve the reproducibility of the work that we publish. This form provides structure for consistency and transparency in reporting. For further information on Nature Research policies, see Authors & Referees and the Editorial Policy Checklist .

Statistics

For all statistical analyses, confirm that the following items are present in the figure legend, table legend, main text, or Methods section.

n/a	Confirmed	
☐	☒	The exact sample size (*n*) for each experimental group/condition, given as a discrete number and unit of measurement
☐	☒	A statement on whether measurements were taken from distinct samples or whether the same sample was measured repeatedly
☐	☒	The statistical test(s) used AND whether they are one- or two-sided<br>*Only common tests should be described solely by name; describe more complex techniques in the Methods section.*
☐	☒	A description of all covariates tested
☐	☒	A description of any assumptions or corrections, such as tests of normality and adjustment for multiple comparisons
☐	☒	A full description of the statistical parameters including central tendency (e.g. means) or other basic estimates (e.g. regression coefficient) AND variation (e.g. standard deviation) or associated estimates of uncertainty (e.g. confidence intervals)
☐	☒	For null hypothesis testing, the test statistic (e.g. *F*, *t*, *r*) with confidence intervals, effect sizes, degrees of freedom and *P* value noted<br>*Give P values as exact values whenever suitable.*
☒	☐	For Bayesian analysis, information on the choice of priors and Markov chain Monte Carlo settings
☐	☒	For hierarchical and complex designs, identification of the appropriate level for tests and full reporting of outcomes
☐	☒	Estimates of effect sizes (e.g. Cohen's *d*, Pearson's *r*), indicating how they were calculated

Our web collection on statistics for biologists contains articles on many of the points above.

Software and code

Policy information about availability of computer code

Data collection	The base editing deep sequencing data was obtained using a MiSeq sequencer that uses proprietary Illumina software to generate fastq files. The high throughput sequencing data for the genome-wide screen was collected using a HiSeq 2500 sequencer that uses proprietary Illumina software to generate fastq files.
Data analysis	All data analysis tools used in the study are open source. Data analysis was performed using Python 2.7 or 3.6 and the Jupyter notebook/lab. A list of the python packages and published command line software used for analysis is provided as Supplementary table 9 in the manuscript. The base editing deep sequencing data was obtained using a MiSeq sequencer that uses proprietary Illumina software to generate fastq files. The high throughput sequencing data for the genome-wide screen was collected using a HiSeq 2500 sequencer that uses proprietary Illumina software to generate fastq files. The following programs were during data analysis: PANDA-Seq v2.11, Needle (EMBOSS v6.6.0.0), Bowtie v1.2.1.1, Rate4site 3.0.0, and FASTX v36.3.8. The following packages for Python 2.7 were used: pandas 0.23.4, matplotlib v2.2.3, numpy v1.15.4, scipy v1.1.0, seaborn v0.9.0, tqdm v4.32, and Biopython v1.71. For Python 3.6, the following packages were used: pandas v0.24.2, numpy v1.16.2, matplotlib v3.0.3, scipy v1.2.1 and seaborn v0.9.0. The code is available at https://github.com/Landrylab/Despres_et_al_2020

For manuscripts utilizing custom algorithms or software that are central to the research but not yet described in published literature, software must be made available to editors/reviewers. We strongly encourage code deposition in a community repository (e.g. GitHub). See the Nature Research guidelines for submitting code & software for further information.

Data

Policy information about availability of data

All manuscripts must include a data availability statement. This statement should provide the following information, where applicable:

- Accession codes, unique identifiers, or web links for publicly available datasets
- A list of figures that have associated raw data
- A description of any restrictions on data availability

All raw sequencing data has been deposited on the NCBI as accession number PRJNA552472. The gRNA screen scores, predicted mutation outcomes, mutation

effect predictors scores, as well as other relevant annotations are provided in Supplementary Dataset 1. Source image files for the tetrad dissections are presented as Supplementary Image 1 and 2.The source data for Figure 4B and Figure 5A is provided as Supplementary table 1 and Supplementary tables 2, 3 and 4. Data on yeast gene essentiality was obtained from http://www-sequence.stanford.edu/group/yeast_deletion_project/. The mutfunc database is available at http://ftp.ebi.ac.uk/pub/databases/mutfunc/mutfunc_v1/yeast/. The Envision database is available at https://envision.gs.washington.edu/shiny/envision_new/. The Uniprot database can be found at https://www.uniprot.org/. The Saccharomyces database can be accessed at https://www.yeastgenome.org/. The PANTHER gene ontology tool can be accessed at http://pantherdb.org/. Phylome DB V4 can be accessed at http://phylomedb.org/. The data from the 1002 yeast genome project can be accessed at http://1002genomes.u-strasbg.fr/files/ allReferenceGenesWithSNPsAndIndelsInferred.tar.gz.

Field-specific reporting

Please select the one below that is the best fit for your research. If you are not sure, read the appropriate sections before making your selection.

- [x] Life sciences
- [ ] Behavioural & social sciences
- [ ] Ecological, evolutionary & environmental sciences

For a reference copy of the document with all sections, see nature.com/documents/nr-reporting-summary-flat.pdf

Life sciences study design

All studies must disclose on these points even when the disclosure is negative.

Sample size	The target read depth for the base editing time course experiment was set to 500-1000 reads per sample, based on known Target-AID editing rates in yeast. Read depth for the The screen was performed in duplicate, with passaging between steps that ensured over 200X theoretical coverage of the gRNA library in the cell population to minimize stochastic effects.
Data exclusions	The difference between the galactose induction time point and the end of the competition experiment were used to measure relative abundance variation and detect gRNAs with fitness effects. A minimal read count threshold at the galactose time point was used to filter out gRNAs with low overall abundance and lower experimental noise. Data from the other time points was used to measure correlation between replicates and assess biases in sequencing based on gRNA GC content, as well as in figure 1D.
Replication	Base editing deep sequencing experiments were performed in two replicates, and show good agreement across different analysis. Screen replicates show good correlation and the read abundances correlation matrixes for experimental timepoints reconstitute the experimental design. (shown in supplementary material). Tetrad dissection was performed with n=12 or n=24 tetrads for confirmation studies.
Randomization	Since the experiment is a time course competition screen, there is no need to randomize groups.
Blinding	Since the experiment is a pooled competition screen, the subjective judgment of the investigator cannot affect results for individual gRNAs.

Reporting for specific materials, systems and methods

We require information from authors about some types of materials, experimental systems and methods used in many studies. Here, indicate whether each material, system or method listed is relevant to your study. If you are not sure if a list item applies to your research, read the appropriate section before selecting a response.

Materials & experimental systems

n/a	Involved in the study	
[x]	[ ]	Antibodies
[x]	[ ]	Eukaryotic cell lines
[x]	[ ]	Palaeontology
[x]	[ ]	Animals and other organisms
[x]	[ ]	Human research participants
[x]	[ ]	Clinical data

Methods

n/a	Involved in the study	
[x]	[ ]	ChIP-seq
[x]	[ ]	Flow cytometry
[x]	[ ]	MRI-based neuroimaging
