# Supplementary material for: Perturbing proteomes at single residue resolution using base editing
Source: Nat Commun. 2020 Apr 20;11:1871. doi: 10.1038/s41467-020-15796-7 (PMC7170841; doi:10.1038/s41467-020-15796-7)

RAP1

MoBY WT MoBY empty

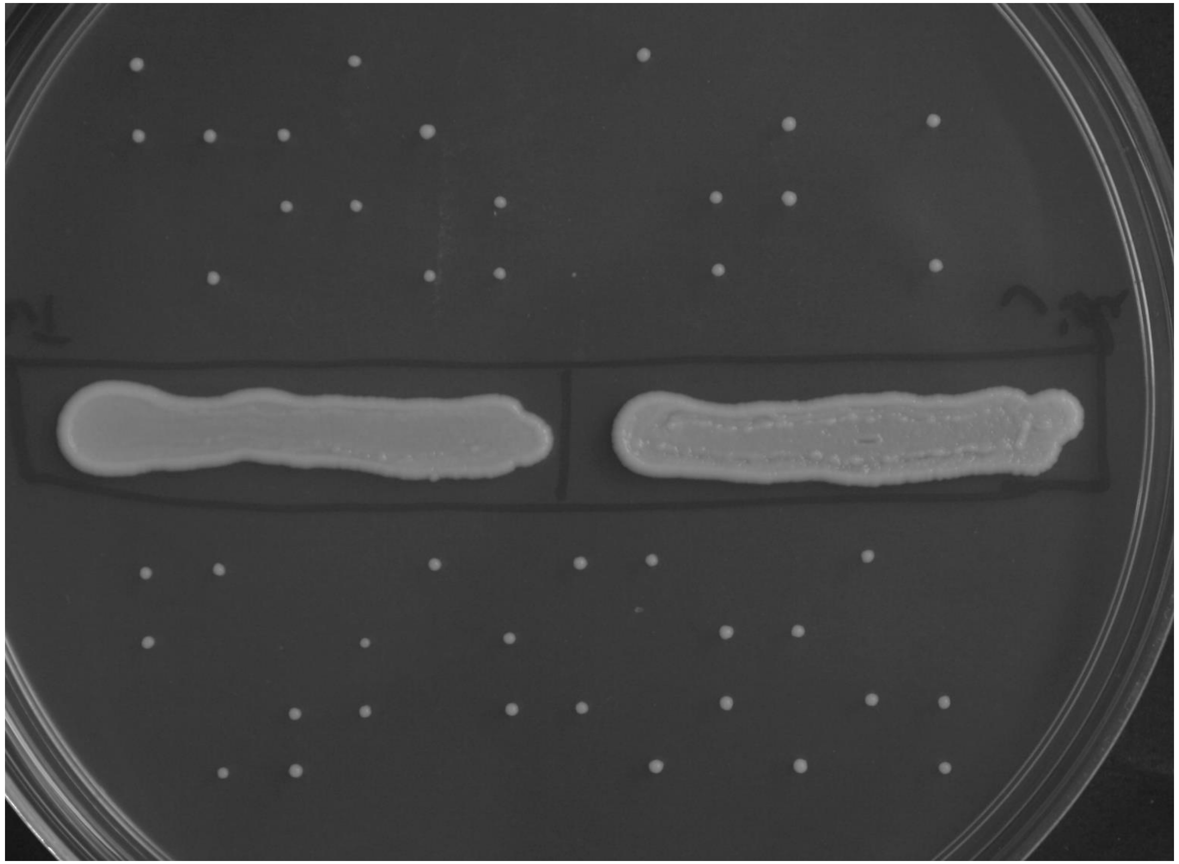

GLN4

MoBY WT

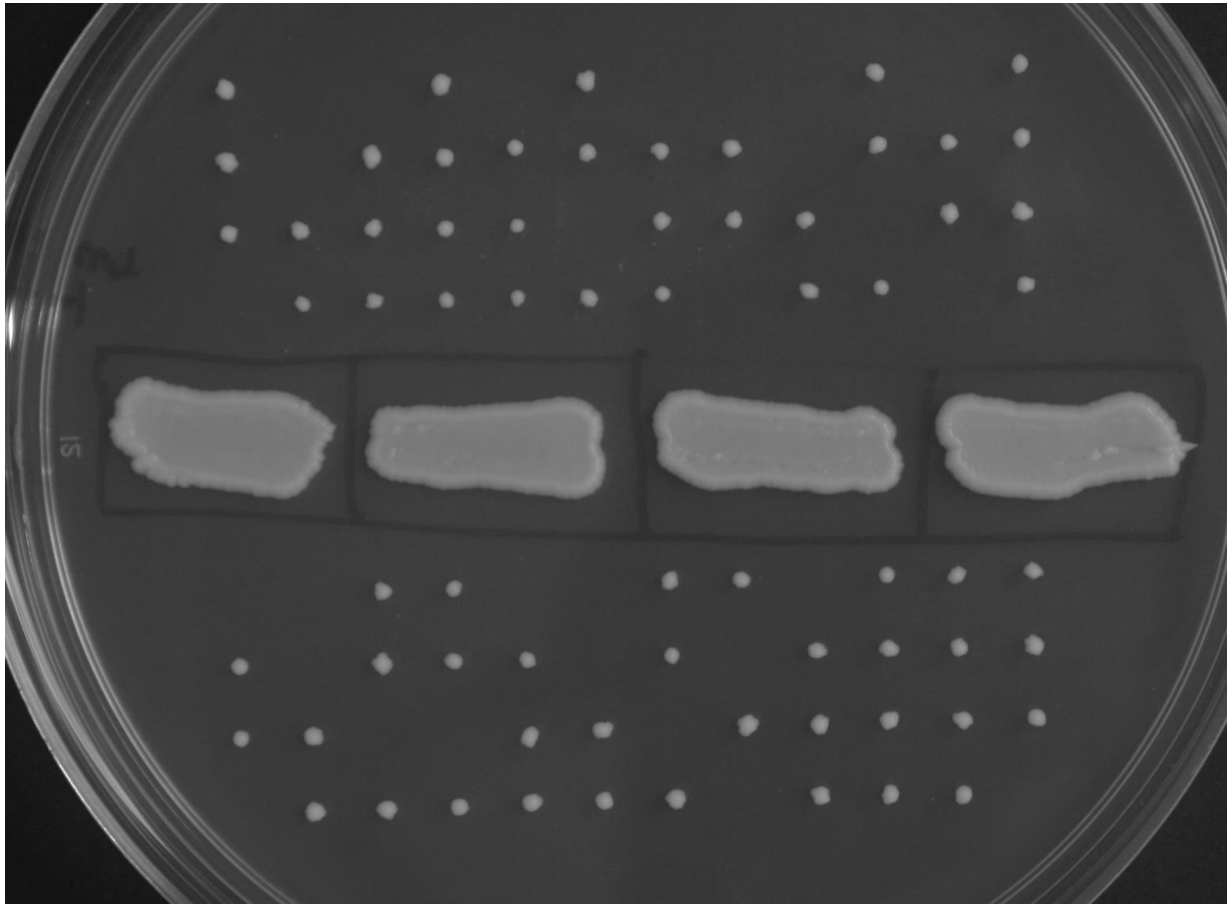

MoBY empty

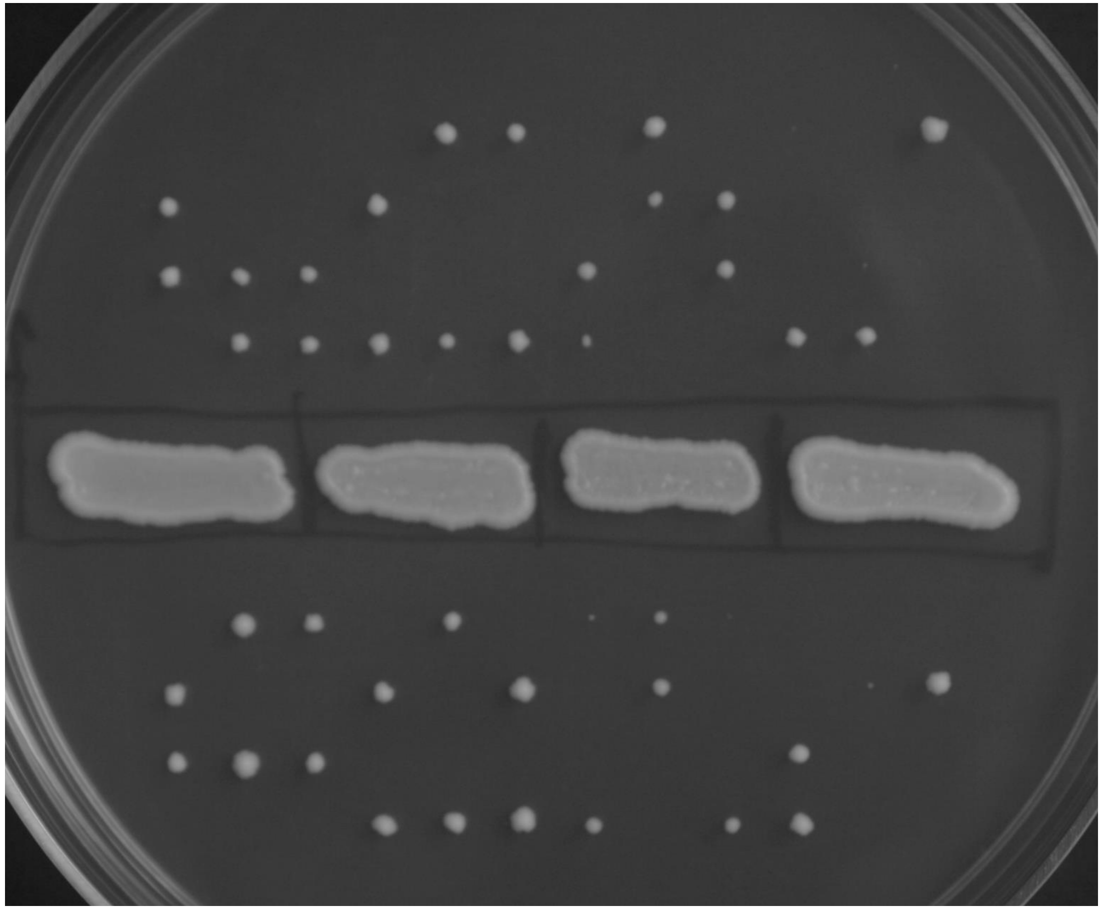

GLN4

G267R

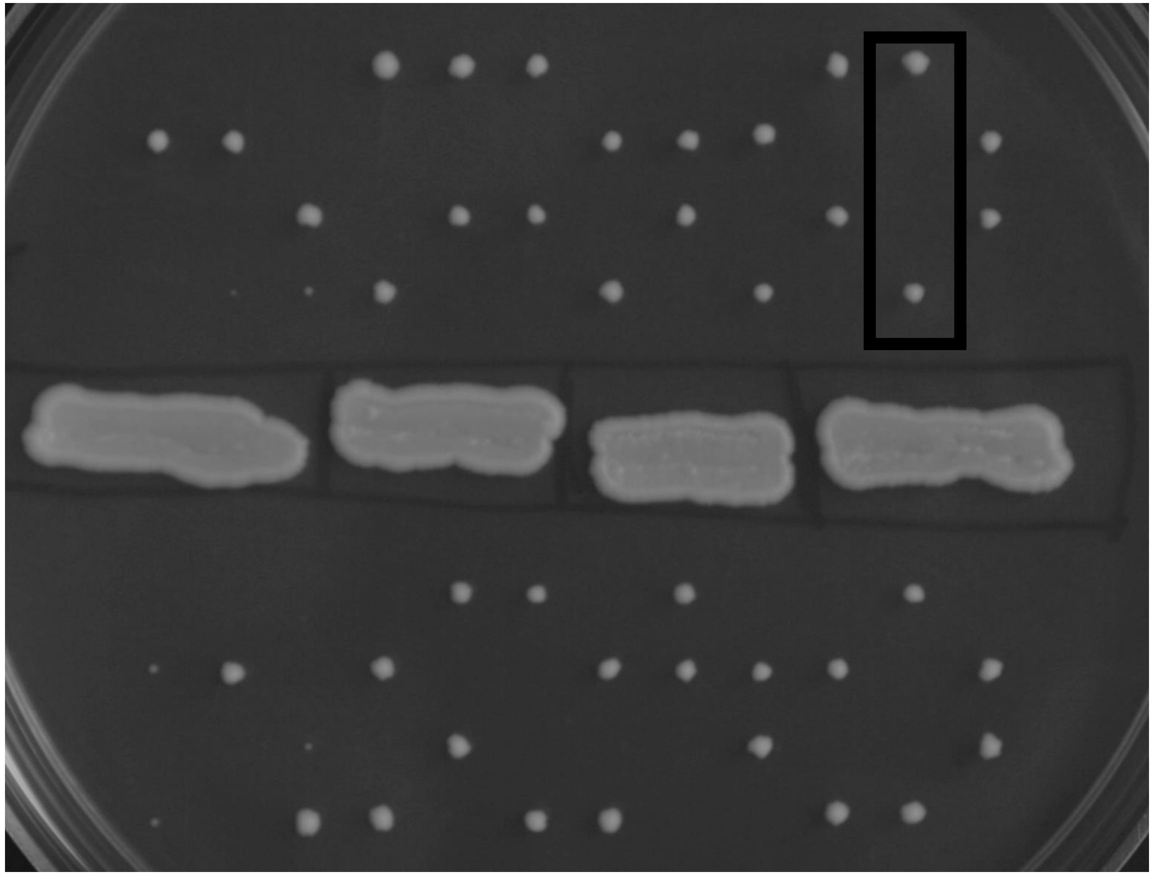

G267S

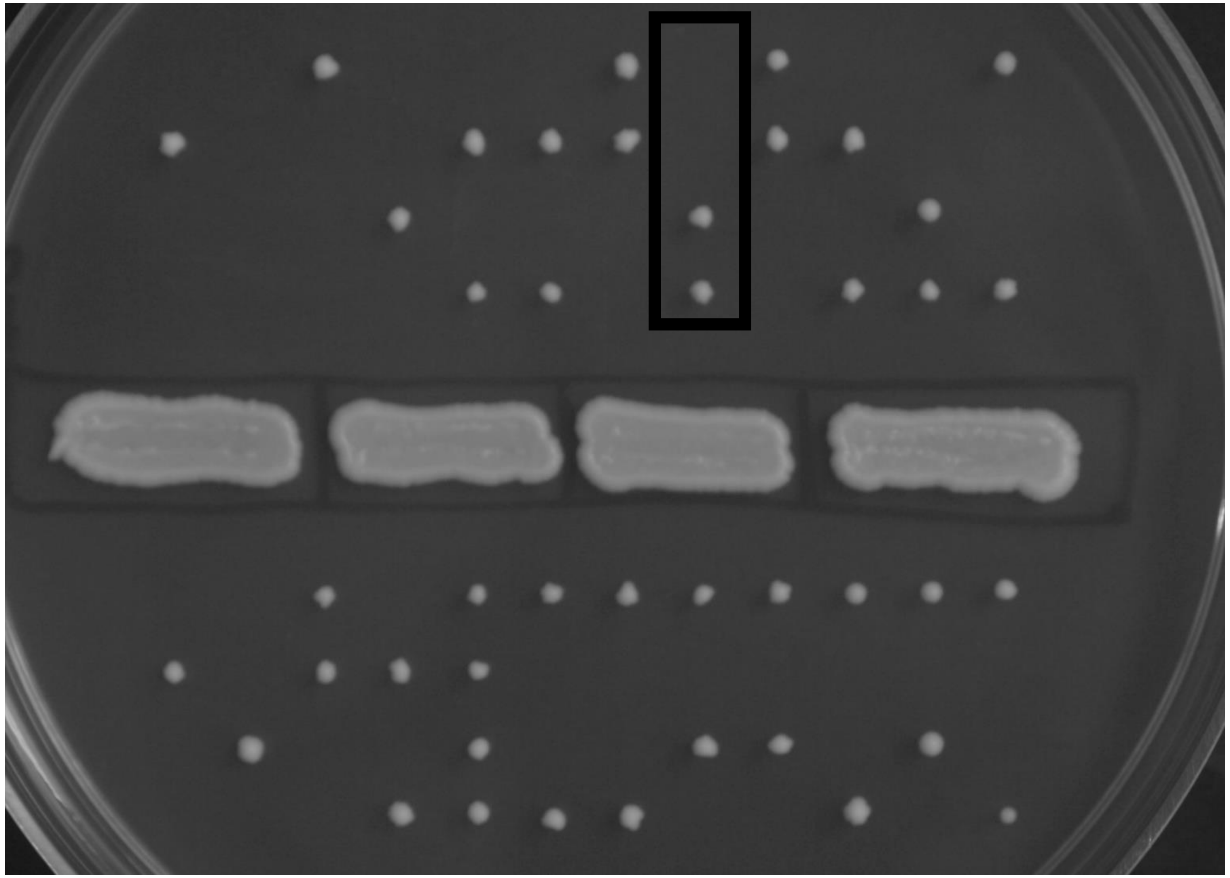

GLN4

D291E D291D

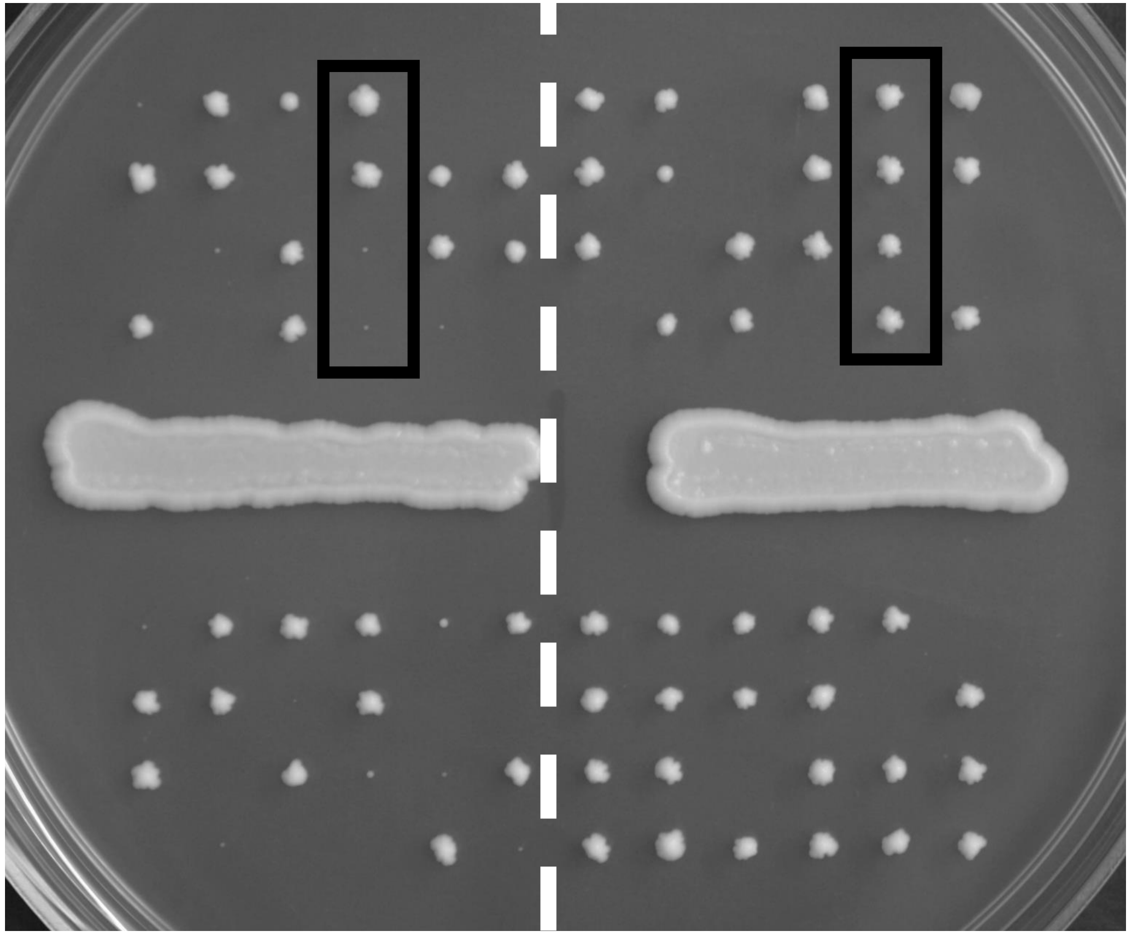

R402T R402K

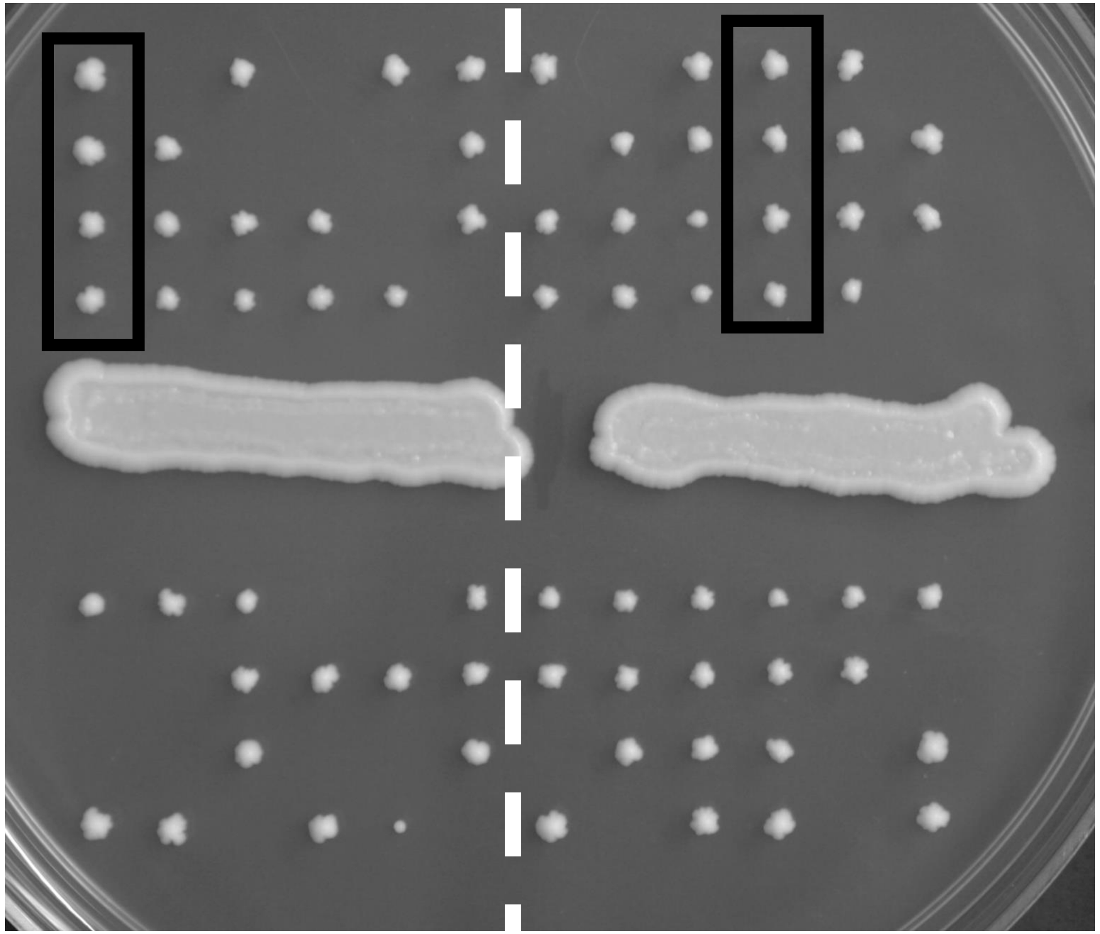

GLN4

R499T R499K T548R T548I

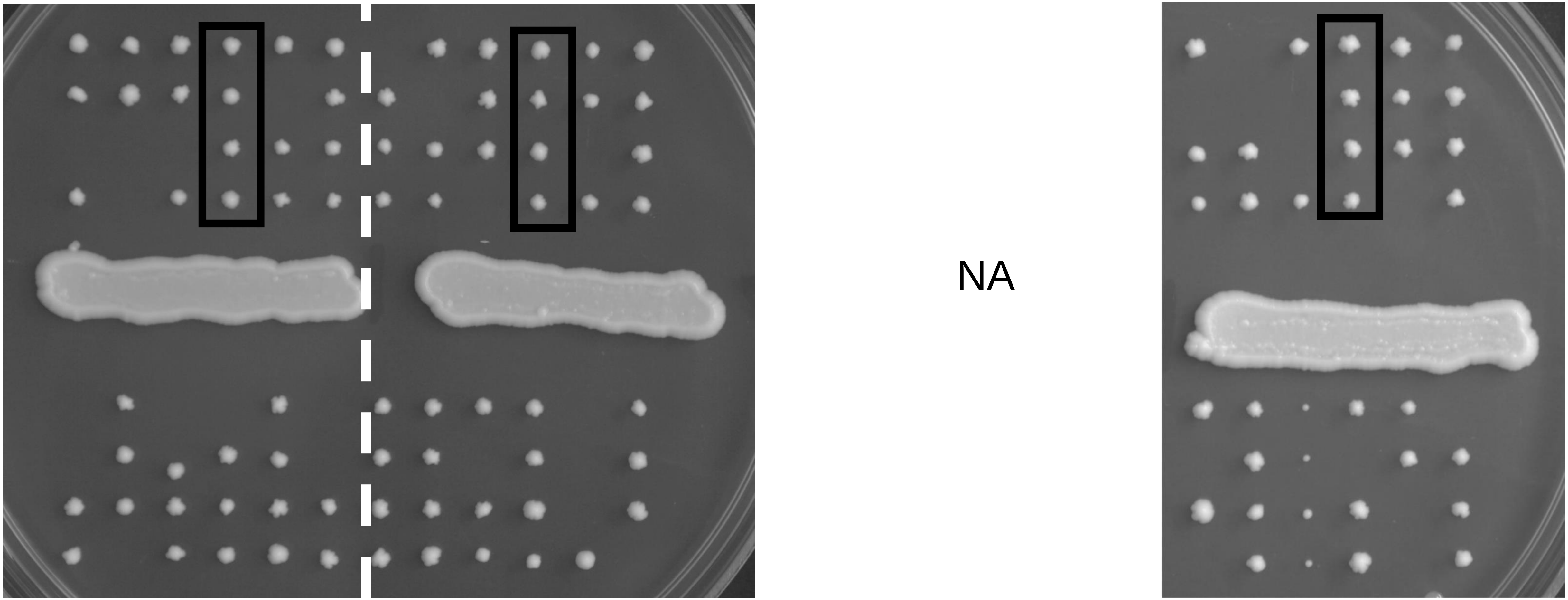

NA

GLN4

R569T

R569K

E301Q

E301R

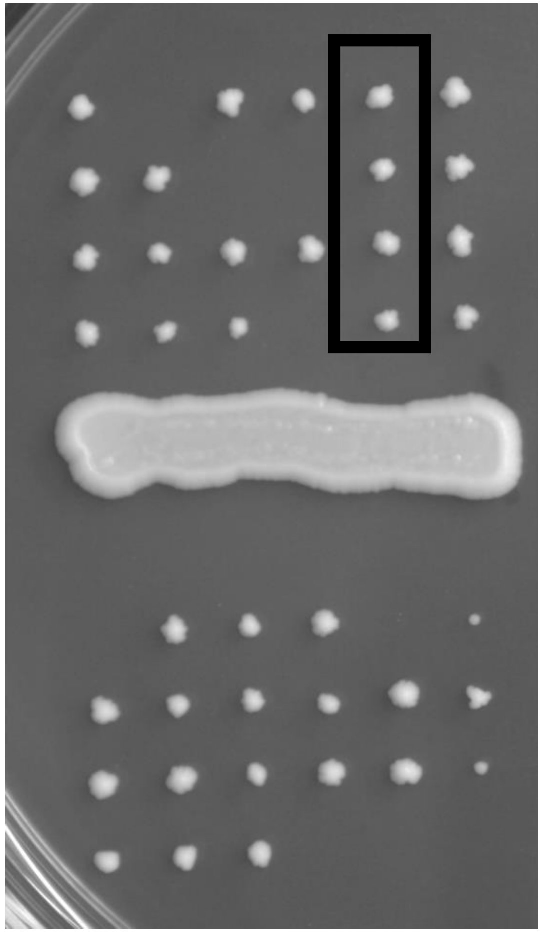

NA

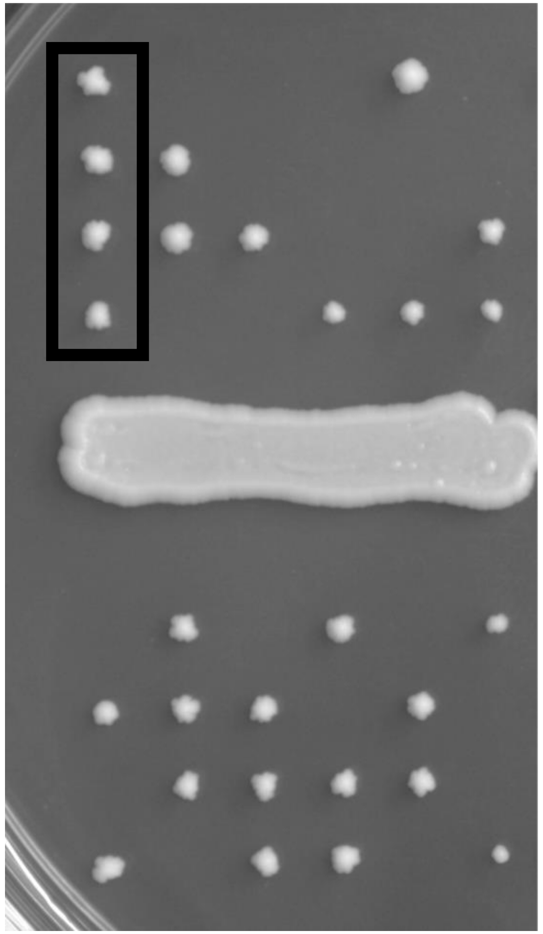

NA

GLN4

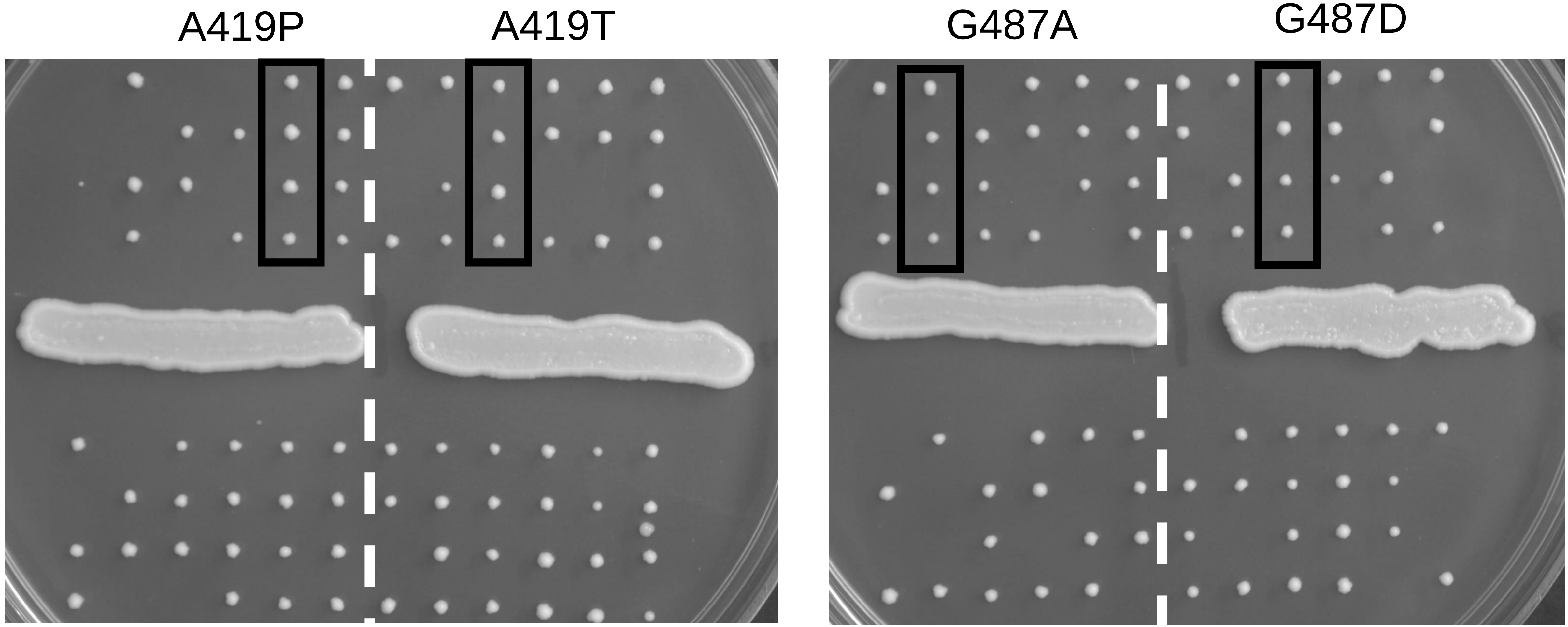

GLN4

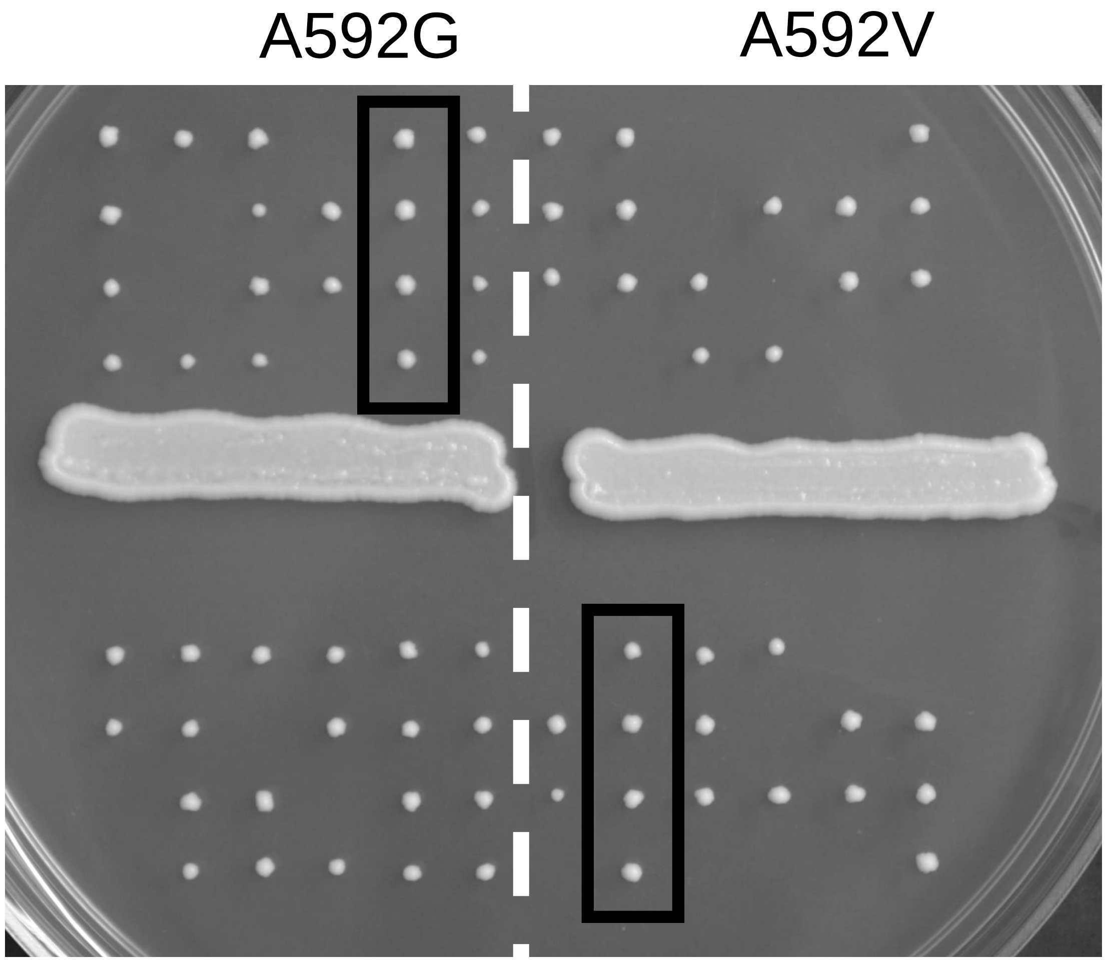

Supplement: Supplementary file 7 — Source Data [file 41467_2020_15796_MOESM7_ESM.zip › SourceData/Source_Image_File_1.pdf]
