# Supplementary material for: Perturbing proteomes at single residue resolution using base editing
Source: Nat Commun. 2020 Apr 20;11:1871. doi: 10.1038/s41467-020-15796-7 (PMC7170841; doi:10.1038/s41467-020-15796-7)
Supplement: Supplementary file 7 — Source Data [file 41467_2020_15796_MOESM7_ESM.zip › SourceData/Source_Image_File_2.pdf]

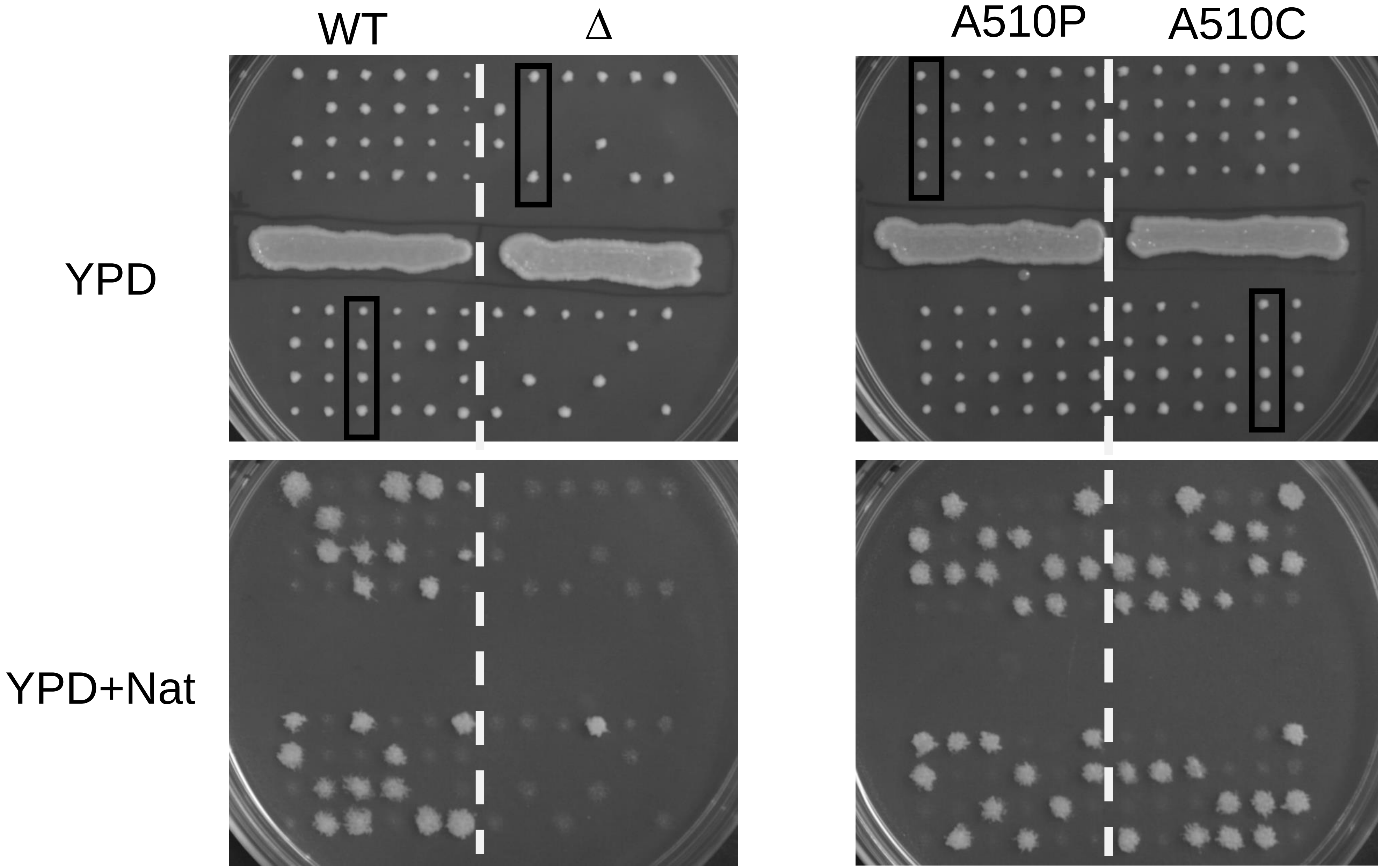

WT
Δ
A510P
A510C
YPD
YPD+Nat

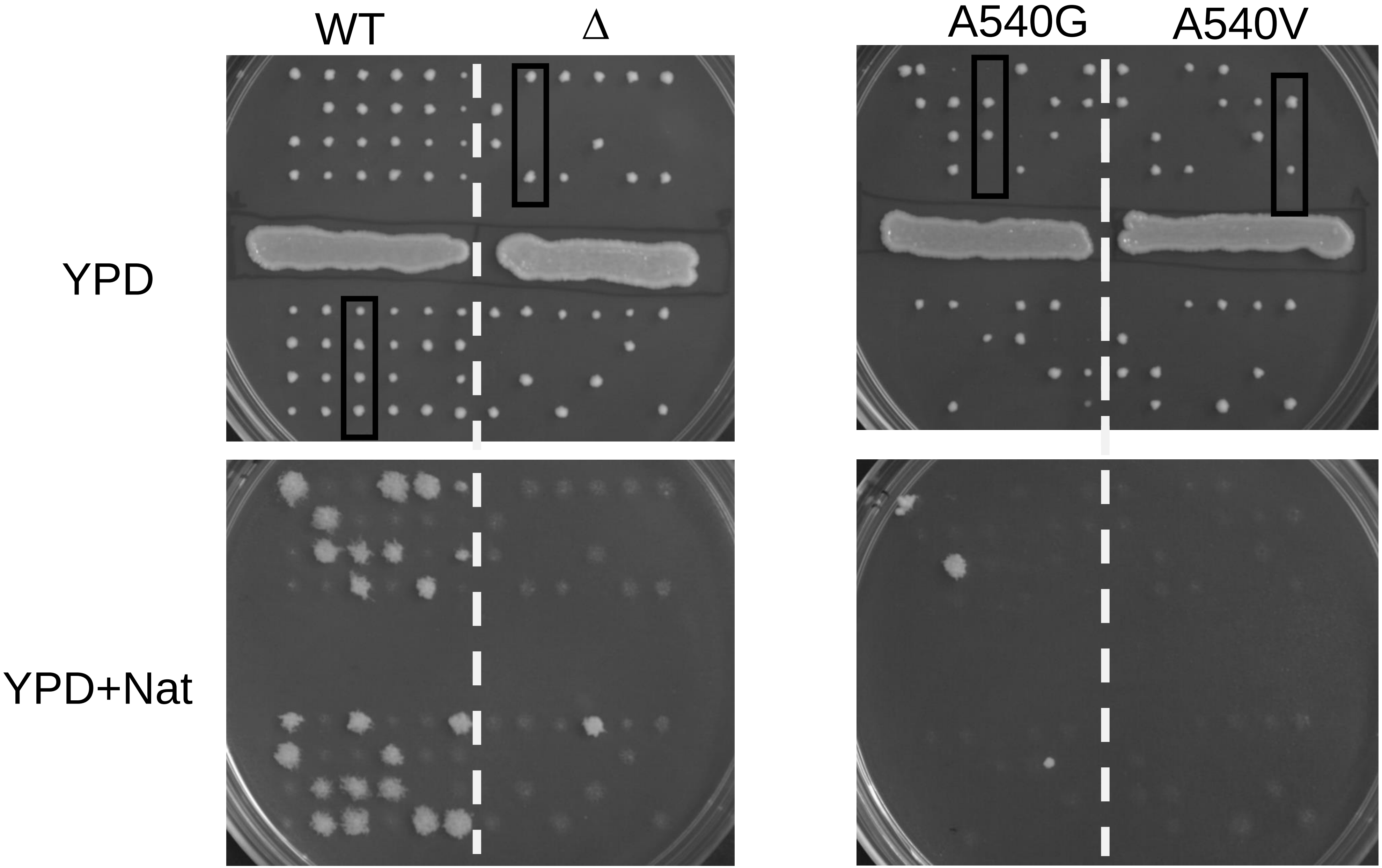
WT
Δ
A540G
A540V
YPD
YPD+Nat

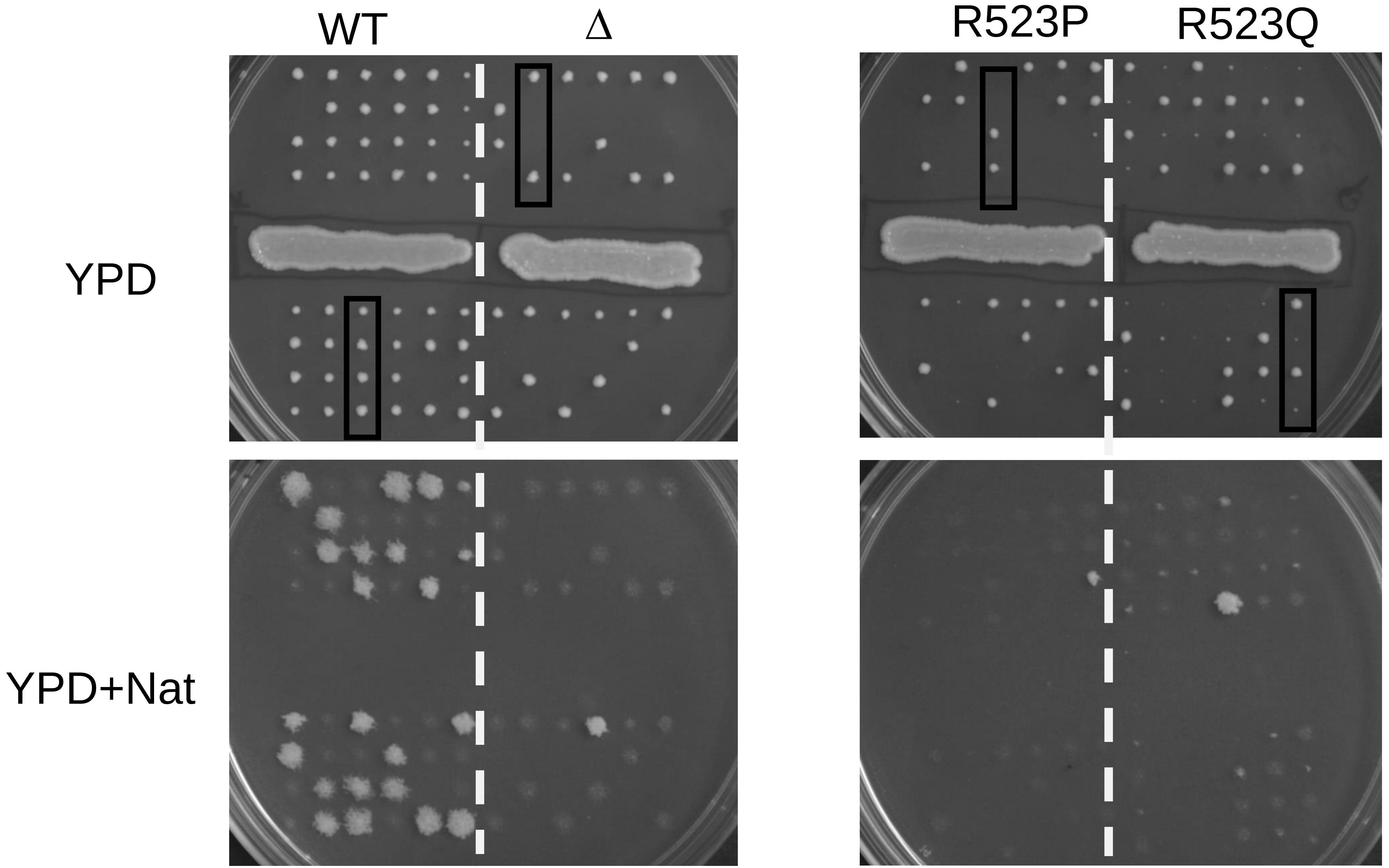
WT
Δ
R523P
R523Q
YPD
YPD+Nat

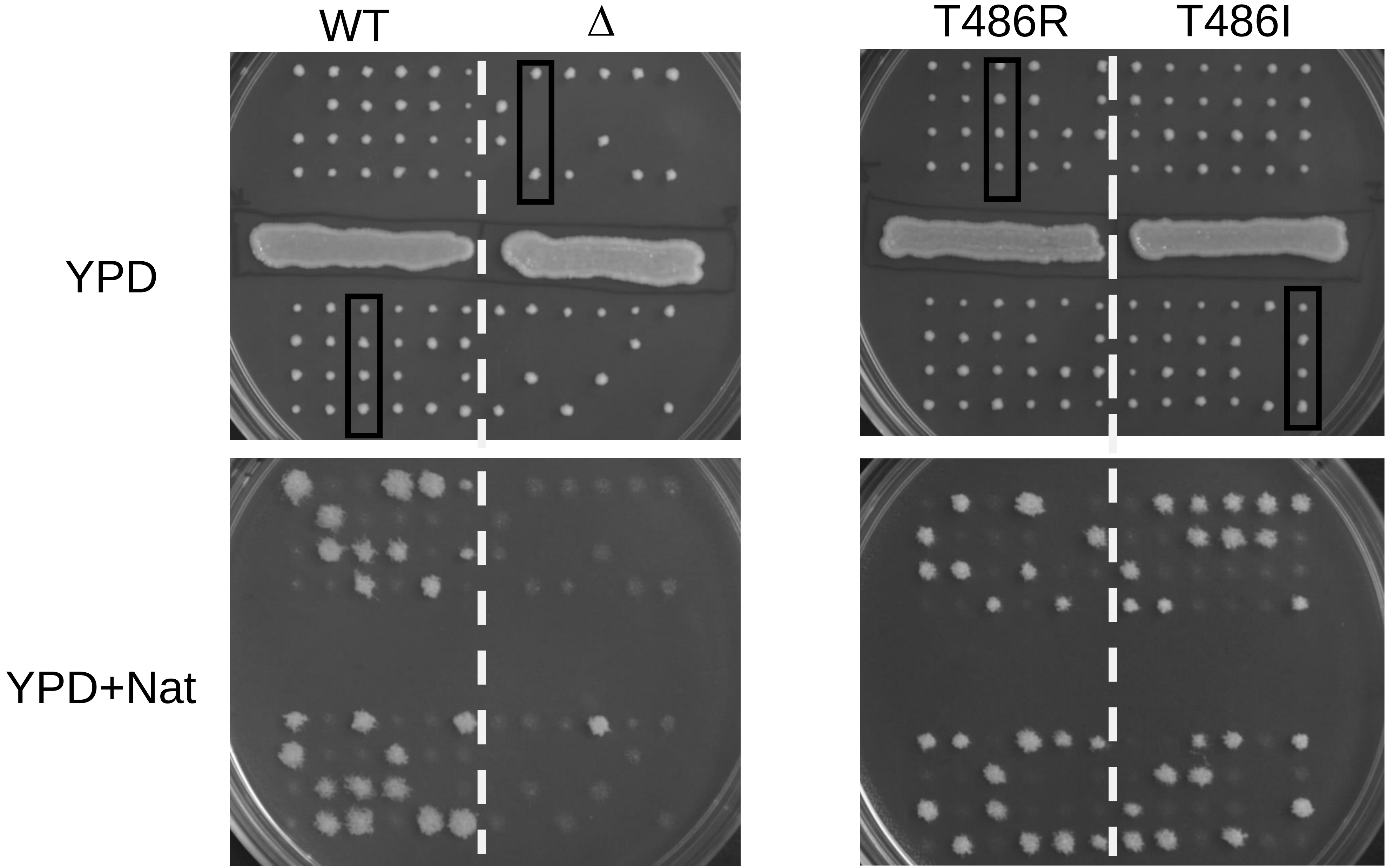
WT
Δ
T486R
T486I
YPD
YPD+Nat

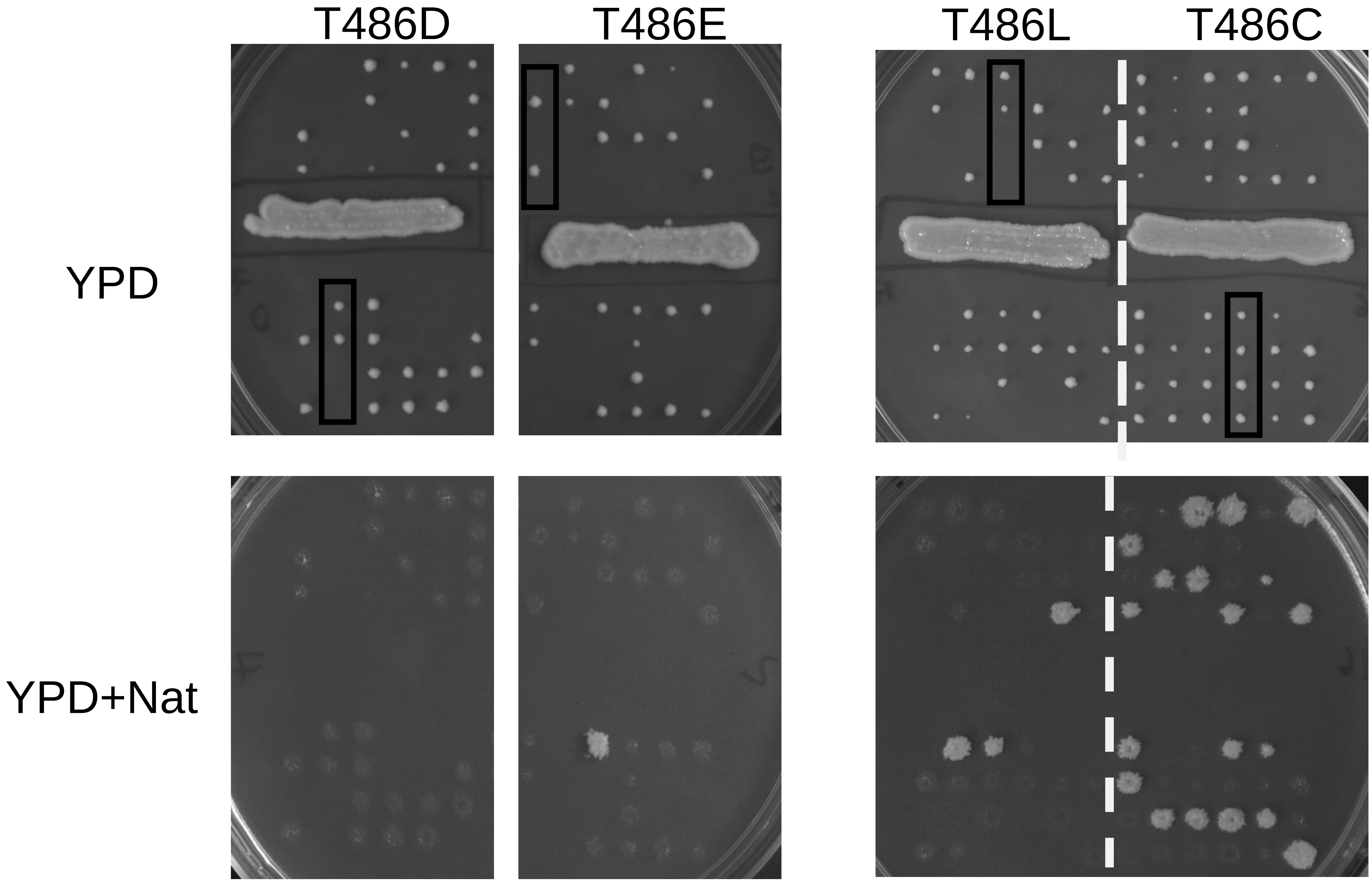

T486D
T486E
T486L
T486C
YPD
YPD+Nat

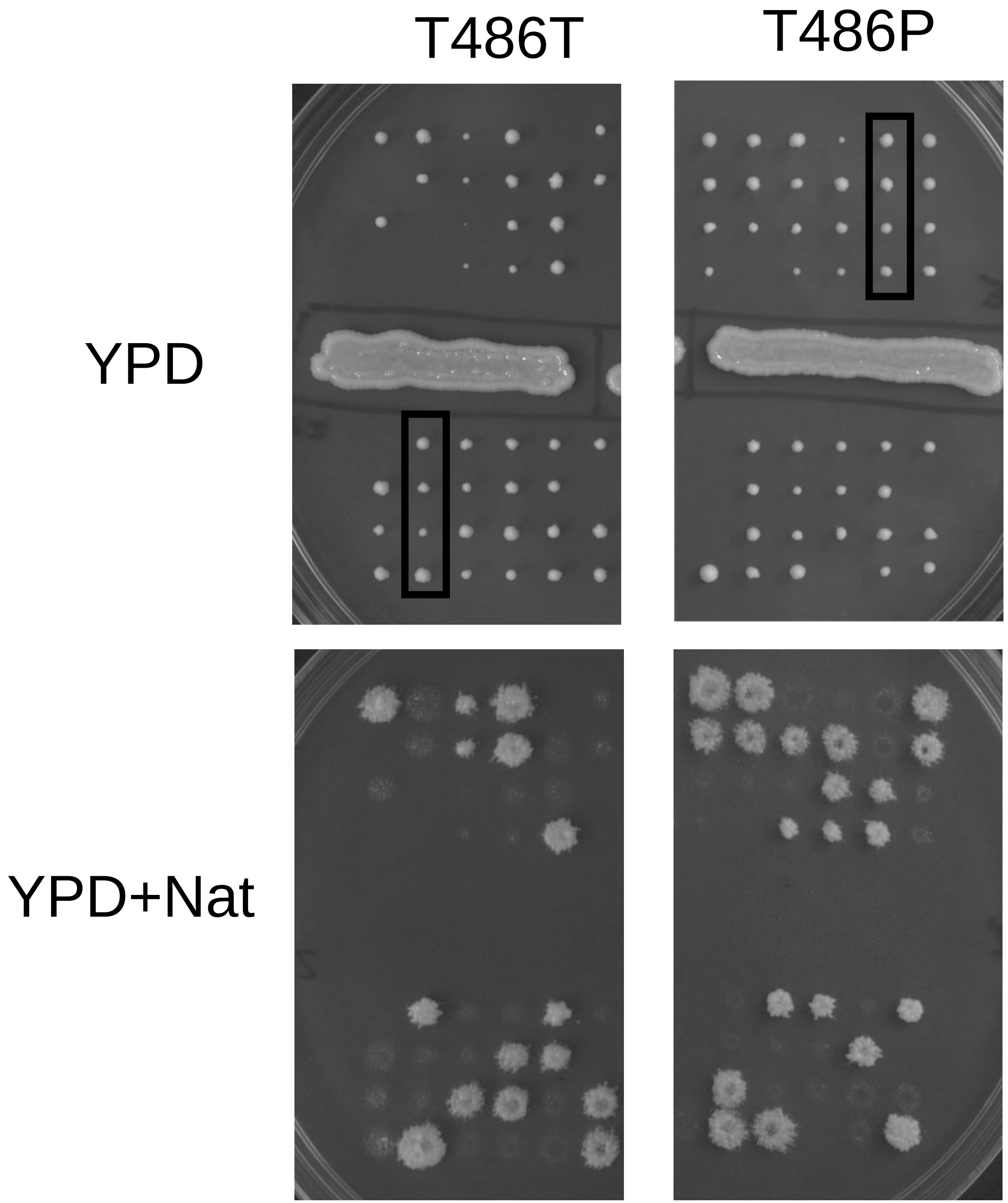
T486T
T486P
YPD
YPD+Nat
